# The Power of *Kawaii*: Viewing Cute Images Promotes a Careful Behavior and Narrows Attentional Focus

**DOI:** 10.1371/journal.pone.0046362

**Published:** 2012-09-26

**Authors:** Hiroshi Nittono, Michiko Fukushima, Akihiro Yano, Hiroki Moriya

**Affiliations:** Graduate School of Integrated Arts and Sciences, Hiroshima University, Higashi-Hiroshima, Hiroshima, Japan; University of Leicester, United Kingdom

## Abstract

*Kawaii* (a Japanese word meaning “cute”) things are popular because they produce positive feelings. However, their effect on behavior remains unclear. In this study, three experiments were conducted to examine the effects of viewing cute images on subsequent task performance. In the first experiment, university students performed a fine motor dexterity task before and after viewing images of baby or adult animals. Performance indexed by the number of successful trials increased after viewing cute images (puppies and kittens; *M* ± *SE* = 43.9±10.3% improvement) more than after viewing images that were less cute (dogs and cats; 11.9±5.5% improvement). In the second experiment, this finding was replicated by using a non-motor visual search task. Performance improved more after viewing cute images (15.7±2.2% improvement) than after viewing less cute images (1.4±2.1% improvement). Viewing images of pleasant foods was ineffective in improving performance (1.2±2.1%). In the third experiment, participants performed a global–local letter task after viewing images of baby animals, adult animals, and neutral objects. In general, global features were processed faster than local features. However, this global precedence effect was reduced after viewing cute images. Results show that participants performed tasks requiring focused attention more carefully after viewing cute images. This is interpreted as the result of a narrowed attentional focus induced by the cuteness-triggered positive emotion that is associated with approach motivation and the tendency toward systematic processing. For future applications, cute objects may be used as an emotion elicitor to induce careful behavioral tendencies in specific situations, such as driving and office work.

## Introduction

Cute things are popular worldwide. In particular, Japan’s culture accepts and appreciates childishness at the social level [1]. Various kinds of anime and character goods, such as *Pokémon* and *Hello Kitty*, which are often described as *kawaii*, are produced and exported to many countries. This phenomenon attracts considerable attention from various fields, including aesthetics [2] and engineering [3]. *Kawaii* is an attributive adjective in modern Japanese and is often translated into English as “cute.” However, this word was originally an affective adjective derived from an ancient word, *kawa-hayu-shi*, which literally means face (*kawa*)-flushing (*hayu-shi*). The original meaning of “ashamed, can’t bear to see, feel pity” was changed to “can’t leave someone alone, care for” [4]. In the present paper, we call this affective feeling, typically elicited by babies, infants, and young animals, cute.

Cute objects are assumed to be characterized by baby schema. This is a set of features that are commonly seen in young animals: a large head relative to the body size, a high and protruding forehead, large eyes, and so forth. Lorenz [5] assumed that responses to baby schema are innate processes and are triggered by elemental features of the stimuli. In humans, the stimuli are deemed cute [6], [7], capture attention [8], [9], bring a smile to the viewer’s face [10], [11], and induce motivation and behavior for approach and caregiving [7], [12], [13]. Baby schema modulates perception and attention at early stages of visual processing [14] and activates the reward system of the brain [15]. From an ethological perspective, it is understandable that cute things are treated favorably. However, little is known about whether encountering a cute object influences the subsequent behavior of the beholder. Because cute things produce positive feelings, their influence may extend to other aspects of behavior.

Sherman, Haidt, and Coan [16] reported two experiments showing that performance in a fine motor dexterity task (the children’s game *Operation*) improved after participants viewed a slide show of cute images (e.g., puppies and kittens) more than after they viewed images that were not as cute (e.g., dogs and cats). The performance measure was the number of plastic body parts that participants removed successfully from the body of the patient depicted on the game board using tweezers without touching the edges of the compartments. The improvement in the accuracy of this task can be interpreted as an index of increased attention to and control of motor actions. Sherman et al. [16] explained this effect in terms of the embodied cognition perspective. That is, the tenderness elicited by cute images is more than just a positive affective feeling state. It can make people more physically tender in their motor behavior. Although the results are intriguing, the mechanism of performance improvement remains unclear for two reasons. First, the time to complete the task was not measured. Better performance could be achieved either through slow and deliberate actions or through quick and accurate actions. Measuring the performance speed would help to explain the underlying mechanism. Second, only one type of task was used. If viewing baby animals induced a behavioral tendency toward protection and caregiving, performance improvement could be specific to a care-related task. The operation task used by Sherman et al. [16] suggests caregiving because the player is expected to act as a doctor who helps the patient depicted on the game board with removing foreign objects from the patient’s body. Using different types of tasks would elucidate the cause of performance improvement. Recently, Sherman and Haidt [17] challenged the classic view that cuteness is an innate releaser of parental instincts and caregiving responses. Instead, they proposed that perceiving cuteness motivates social engagement and primes affiliative, friendly tendencies. This attitudinal change is assumed to be linked with cognitive processes related to mentalizing (i.e., attributing mental states to agents) and sometimes indirectly leads to increased cares. If cuteness-induced behavioral carefulness is caused by a heightened motivation for social interaction, the effect would not be found in simple perceptual–cognitive tasks that do not suggest social interaction.

The present study aimed to examine the effect of viewing cute images of baby animals on subsequent performance in three behavioral experiments. In Experiment 1, the same operation task from Sherman et al. [16] was used. The changes within the completion time for the task as well as the performance score before and after viewing cute images were examined. The prediction was that images of baby animals would be rated as cuter than images of adult animals and that the participants’ performance would improve more after viewing the cute images. If this result was due to a behavioral shift toward slow and deliberate actions, the time to complete the task would be extended. In Experiment 2, whether the effect is specific to a motor task that suggests helping others was examined by using a non-motor visual search task that required concentration but did not involve fine motor skills nor suggest social interaction. In Experiment 3, further investigation was conducted on whether viewing cute images leads to the change in attentional focus in a global–local letter task [18], [19].

To examine possible gender differences, both female and male participants were included in the experiments. Previous research reported that women are more interested in infants [20] and have greater ability to discriminate cuteness cues in them [21], [22] than men. In contrast, behavioral studies often failed to find significant gender differences in attention capture [8], subsequent motor performance [16], and viewing time adjustment [23] in response to infant stimuli. It was predicted that women could be more reactive to experimental manipulation in the present study, but the gender effect, if any, would be weak.

## Experiment 1

### Ethics Statement

The protocols of the three experiments were approved by the Research Ethics Committee of the Graduate School of Integrated Arts and Sciences in Hiroshima University. All participants provided written informed consent. After the experiment, participants were debriefed about the purpose of the study. Participants gave permission to use their data in the analysis.

### Materials and Methods

Forty-eight university students (24 men and 24 women, 18–22 years old, *M* = 18.6) were randomly assigned to one of two conditions: *baby animal* condition or *adult animal* condition (*n* = 24, 12 men and 12 women). Participants were all right-handed according to the Edinburgh Handedness Inventory [24]. The participants were asked to perform a task requiring a high level of concentration twice, with a break of a few minutes between the tasks for mental recovery. To use this spare time beneficially, the participants were asked to help select stimulus images for another experiment. All participants agreed.

The task was a children’s game (*Bilibili Dr. game*, Megahouse, Tokyo, Japan) similar to that used by Sherman et al. [16]. Using tweezers, the participants were asked to remove 14 small pieces from holes on the patient’s body depicted on the game board without touching the edges of the holes. Dropping a piece while attempting to remove it was considered a failure. After watching a demonstration by the experimenter, the participants were instructed to perform the task at their own pace with the goal of obtaining the highest score possible. The participants started the task on the experimenter’s cue. The holes were sequentially numbered. After each trial, regardless of success or failure, the participants immediately moved on to the next hole. When the participants finished all the 14 trials, the task was completed. The number of successful removals and the time to complete the task were recorded.

Between the two sessions, participants were given seven sheets of cardboard (18×25 cm) with a color photo image of an animal printed on each sheet. They were asked to rank the images in 1.5 minutes according to their preference. Seven images of puppies and kittens were used for the baby animal condition, and seven images of dogs and cats were used for the adult animal condition. These images were selected in a pilot survey from 30 images that included the images used by Sherman et al. [16] and similar royalty-free images downloaded from the Internet. The images of baby and adult animals were selected so that they differed in cuteness and infantility ratings but did not differ in pleasantness and excitement ratings. At the end of the experiment, each participant saw the seven pictures he or she sorted during the break and rated them on four 6-point scales (cute, infantile, pleasant, and exciting; from 1 = *not at all* to 6 = *very much*). The interval between the end of the first session and the start of the second session was less than four minutes and almost constant across participants.

The time to complete the task (in seconds) was logarithmically transformed to meet the criteria for normality. The performance score and time were submitted to a mixed-design analysis of variance (ANOVA) with a within-participants factor of session (pre vs. post) and between-participants factors of condition and gender. For each individual participant, the mean rating scores of the seven images he or she viewed were calculated. The rating scores were compared between groups by *t* tests (two-tailed). Pearson’s correlation coefficients between the rating scores and the performance improvement (post minus pre) were then computed across all participants. The significance level was set at 5% for all analyses. The effect sizes for *t* tests were shown using the point biserial correlation coefficient (*r*). The effect sizes for ANOVAs were shown using the partial η^2^ (η_p_^2^).

### Results and Discussion

Table 1 summarizes the mean rating scores in Experiment 1, along with those in the other experiments. The images of baby animals were rated as cuter (*M* = 4.8 vs. *M* = 4.2) and more infantile (*M* = 4.8 vs. *M* = 2.7) than the images of adult animals, *F*s(1, 44) = 7.31 and 117.35, *p* = 0.010 and *p*<0.001, η_p_^2^s = 0.14 and 0.73, respectively. No significant differences were found in the pleasantness and excitement ratings (*M*s = 4.5 and 4.4 and *M*s = 2.0 and 2.0, respectively), *F*s <1.

**Table 1:** Means and standard deviations of subjective rating scores on the images.

		Baby animals		Adult animals		Control*	
		*M*	*SD*	*M*	*SD*	*M*	*SD*
Experiment 1	Cute	4.8_a_	0.6	4.2_b_	0.8		
	Infantile	4.8_a_	0.7	2.7_b_	0.6		
	Pleasant	4.5	0.7	4.4	0.8		
	Exciting	2.0	1.0	2.0	0.9		
Experiment 2	Cute	5.0_a_	0.3	3.8_b_	0.4	2.2_c_	0.8
	Infantile	5.0_a_	0.4	2.3_b_	0.7	1.8_b_	0.7
	Pleasant	4.5_a_	0.7	4.2_a_	0.3	5.0_b_	0.5
	Exciting	1.9	0.9	2.1	0.8	2.1	1.0
Experiment 3	Cute	5.0_a_	0.7	3.9_b_	0.6	2.6_c_	0.6
	Infantile	5.0_a_	0.6	2.5_b_	0.7	2.1_c_	0.7
	Pleasant	4.7_a_	0.7	4.1_b_	0.7	3.0_c_	0.7
	Exciting	3.0_a_	1.3	2.8_a_	1.0	1.9_b_	0.9

* Pleasant foods (Experiment 2) or neutral objects (Experiment 3).

Note. Ratings were made on 6-point scales (from 1 = *not at all* to 6 = *very much*). Means in the same row that do not share subscripts differ at *p*<0.05.

**Figure 1:**
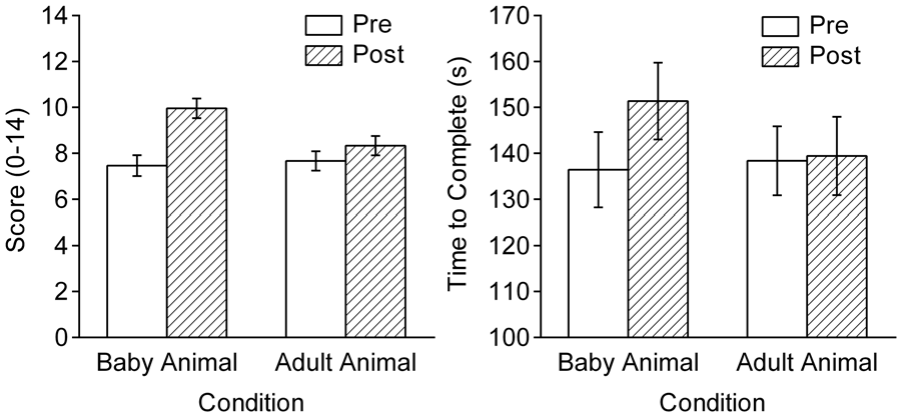
Mean scores and times to complete the operation task (Experiment 1). Participants performed the task before and after viewing images of baby and adult animals (*n* = 24 each). The error bars indicate the standard errors of means.

Figure 1 shows the mean number of successful trials and the mean time to complete the operation task. A Condition×Session×Gender ANOVA showed a significant Condition×Session interaction for both measures, *F*s(1, 44) = 8.93 and 10.62, *p*s = 0.005 and 0.002, η_p_^2^s = 0.17 and 0.19. The group that viewed images of baby animals showed an increase in performance score and in completion time in the second trial, *t*s(23) = 4.83 and 4.93, *p*s <.001, *r*s = 0.51 and 0.19, respectively. No significant increase was found for the group that viewed images of adult animals, *t*s(23) = 2.03 and 0.04, *p*s = 0.054 and 0.972, *r* = 0.16 and *r* <0.01. The performance improvements (*M*±*SE*) were 43.9±10.3% and 11.9±5.5% after viewing images of baby and adult animals. The completion time (in seconds) increased by 12.2±2.6% and 0.8±2.4%, respectively. At the individual level, the increase in performance score was positively correlated with the mean ratings of cuteness and infantility, *r*s(46) = 0.33 and 0.45, *p*s <0.05, respectively. No significant correlations were found for pleasantness and excitement ratings, *r*s = 0.21 and 0.01. No gender-related main and interaction effects were found.

Experiment 1 replicates the results of Sherman et al. [16]. The increase in the performance score was accompanied by an increase in the completion time. This finding suggests that viewing cute images makes participants behave more deliberately and perform tasks with greater time and care.

The results allow more than one interpretation about the underlying mechanism. First, the performance improvement could be due to the slowing down of behavior. For example, research on infant-directed speech has shown that humans (including children) usually talk to babies at a slower tempo than when talking to adults and pronounce individual words more slowly [25], [26]. This behavioral tendency, induced by viewing images of babies, may transfer to subsequent task performance. This tendency may benefit some tasks that require accuracy (e.g., the operation task) but impair other tasks that required speed. If viewing cute images tends to slow behavior, performance on a speeded task would decrease rather than increase. Second, the effect might not be specific to fine motor skills. Caring for babies (nurturance) not only involves tender treatments but also requires careful attention to the targets’ physical and mental states as well as vigilance against possible threats to the targets [17], [27]. If viewing cute things makes the viewer more attentive, the performance of a non-motor perceptual task would also be improved. Third, the effect could be caused by a heightened motivation for social engagement induced by viewing cute images [17]. The performance in the operation task might be improved because it is a care-related task that suggests helping others. If so, the effect would not occur for a task without a hint of social interaction.

To answer these questions, a new experiment was conducted to examine the effect of viewing cute images on the performance of a speeded non-motor visual search task. In addition, whether the observed effect was specific to cute images was examined by adding a third condition in which participants viewed highly pleasant (but not cute or infantile) food images. If the effect was due to pleasant feelings in general, performance would also improve after viewing food images.

## Experiment 2

### Materials and Methods

**Figure 2:**
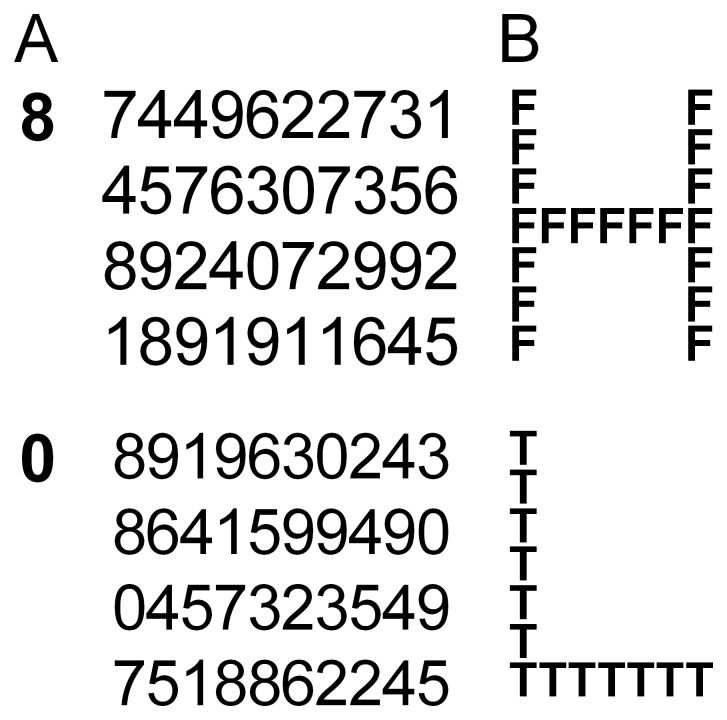
Examples of task stimuli used in Experiments 2 (A) and 3 (B). A: Participants searched a matrix for the designated digit shown on the left side of each matrix and to state the number of counts vocally. The answers are 2 (upper) and 3 (bottom). B: Participants indicated whether a stimulus contained the letter *H* or the letter *T* by pressing the left or right key on a response pad. The upper is a global target stimulus and the bottom is a local target stimulus.

The participants were 48 right-handed university students (24 men and 24 women, 18–20 years old, *M* = 19.0) who did not participate in Experiment 1. Instead of the operation task, these participants performed a visual search task on matrices [28] twice. Figure 2A shows examples of the matrix stimuli. Each matrix consisted of 40 digits (0–9, 4 rows by 10 columns), which were distributed randomly. Ten matrices were printed on one sheet of paper. No two matrices were the same. Participants were asked to search a matrix for the designated digit (shown on the left side of each matrix) without pointing at the digits and to state the number of counts vocally. The range of answers varied from two to six. The target digit differed from one matrix to another. Participants were told to provide as many accurate responses as possible within a time limit of 3 min. Each participant viewed different arrays in two sessions. The other aspects of the procedure were identical to those in Experiment 1, except for the addition of a third group, who viewed images of pleasant foods. Seven food images (e.g., steak, pasta, and sushi) that were rated as highly pleasant in a pilot survey were used. Sixteen participants (8 men and 8 women) were assigned to each condition. The numbers of correct answers and the error rates were submitted to a Condition×Session×Gender ANOVA.

### Results and Discussion

The mean scores of picture ratings are shown in Table 1. The cuteness and infantility ratings differed across conditions, *F*s(2, 42) = 121.31 and 115.19, *p*s <0.001, η_p_^2^s = 0.85 and 0.85. Pleasantness ratings also differed across conditions, *F*(2, 42) = 8.26, *p* = 0.001, η_p_^2^ = 0.28. No significant effect was found for excitement ratings, *F* <1. Post hoc comparisons using *t* tests with the Bonferroni correction showed the following results. The images of baby animals were rated as cuter (*M* = 5.0 vs. *M* = 3.8) and more infantile (*M* = 5.0 vs. *M* = 2.3) than the images of adult animals. These animal images did not differ in pleasantness ratings. The images of pleasant foods were rated as less cute (*M* = 2.2) but more pleasant (*M* = 5.0) compared to the images of baby and adult animals (*M*s = 4.5 and 4.2). Women gave higher cuteness ratings than men to all types of stimuli (*M* = 3.9 vs. *M* = 3.5), *F*(1, 42) = 6.10, *p* = 0.018, η_p_^2^ = 0.13.

**Figure 3:**
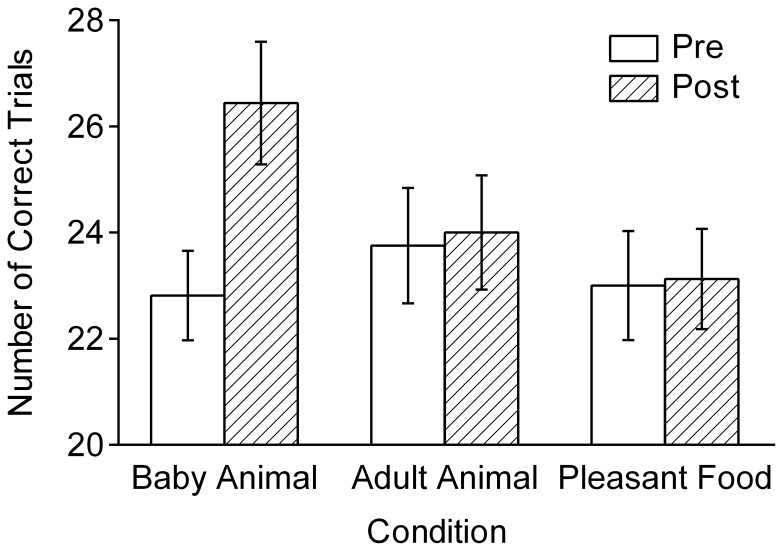
Mean numbers of correct trials in the visual search task (Experiment 2). Participants performed the task before and after viewing images of baby animals, adult animals, and pleasant foods (*n* = 16 each). The error bars indicate the standard errors of means.

Figure 3 shows the mean number of correct answers in the visual search task. The ANOVA showed a significant interaction of condition and session, *F*(2, 42) = 14.64, *p*<0.001, η_p_^2^ = 0.41. Post hoc comparisons showed that the participants who viewed images of baby animals showed a significant improvement in the second trial, *t*(15) = 6.54, *p*<0.001, *r* = 0.42. No improvement was found for the participants who viewed images of adult animals and pleasant foods, *t*s(15) <0.51, *p*s >0.62, *r*s <0.03. The improvements (*M* ± *SE*) were 15.7±2.2%, 1.4±2.1%, and 1.2±2.1% after viewing images of baby animals, adult animals, and pleasant foods, respectively. The increase in the number of correct trials (post minus pre) was positively correlated with the mean cuteness and infantility rating scores of each participant, *r*s(46) = 0.54 and 0.57, *p*s <0.01, respectively. No significant correlations were found for the pleasantness and excitement ratings, *r*s = –0.12 and –0.16. For the error rate (*M* = 9.0%), the Condition×Session interaction was not significant, *F*(2, 42) = 2.08, *p* = 0.138, η_p_^2^ = 0.09. Consistent with the measure of the number of correct trials, the number of searched matrices increased by 13.2±2.2%, 4.3±1.0%, and 3.2±1.9% after viewing images of baby animals, adult animals, and pleasant foods, respectively. The main effect of gender on the number of correct answers was significant, *F*(1, 42) = 4.25, *p = *0.045, η_p_^2^ = 0.09, indicating that female participants performed better than male participants in the visual search task (*M* = 25.0 vs. *M* = 22.7). However, the interaction of gender with condition and session was not significant, *F*(2, 42) = 1.80, *p* = 0.178, η_p_^2^ = 0.08. No other gender-related differences were found.

The results show that viewing cute images improves behavioral performance on a non-motor speeded task. In contrast to Experiment 1, the improvement was associated with faster task performance. Therefore, the effect of viewing cute images on subsequent behavior is not attributable to slower action tendencies toward infants [25], [26]. Because the task does not suggest helping others or any kind of social interactions, it is unlikely that the performance improvement was caused by the heightened sociality motivation that is assumed to be induced by the perception of cuteness [17]. The finding that highly pleasant food images did not affect subsequent performance indicates that the general pleasantness of images is not the main cause of performance improvement.

The results of Experiment 2 extend the previous finding that viewing cute images promotes fine motor dexterity; it also increases carefulness in the perceptual domain. One possible mechanism underlying the effect is that the detection of target digits may be facilitated by narrowing the breadth of attentional focus. That is, the reduction of the interference from surrounding non-target digits should make visual search more efficient. It is known that affective states influence the breadth of attentional focus [29], [30], [31] and that this modulation appears at early visual input stages [32], [33]. Viewing cute images induces positive affective states associated with smiles [10], [11], so that it may modulate one’s focus of attention. Recent studies suggest that positive affect is not a single entity and that different types of positive emotions may have different functions [27], [34]. For instance, Gable and Harmon-Jones [18] focused on the level of approach motivation and proposed that positive affect with high approach motivation (e.g., induced by delicious images of dessert) narrows attentional focus, whereas positive affect with low approach motivation (e.g., induced by entertaining images) broadens attentional focus. In this respect, cute images are considered to induce positive affect with high approach motivation because they are evolutionally related to caregiving and nurturing [5] or because they prime social engagement [17]. Gable and Harmon-Jones [18] used a global–local letter task [19] to examine the effect of viewing pleasant images on the breadth of attentional focus. Generally, people tend to process the global features of a stimulus preferentially compared to the local features [35]. They found that viewing images of baby animals reduces the global precedence effect compared to viewing neutral images of rocks (Study 3 in Ref. [18]).

In Experiment 3, an attempt was made to replicate this finding by comparing the effects of animal images that were rated as high and low in cuteness. It is known that viewing animals (without regard for cuteness) influences human physiological states and social behavior [36], [37]. To confirm that the observed effect is related to cuteness, cute, baby animals were directly compared with less cute, adult animals, as well as neutral objects. It was expected that viewing cute images would narrow the viewer’s attentional focus (and would thus reduce the global precedence effect) compared to viewing images that were less cute or neutral.

## Experiment 3

### Materials and Methods

The experiment was conducted with a within-participants design. The participants were 36 right-handed university students (18 men and 18 women, 18–22 years old, *M* = 20.2) who did not participate in the previous experiments. Under three conditions, the participants performed a reaction time (RT) task after viewing images of baby animals, adult animals, and neutral objects. Eight images were selected for each condition. For the baby animal condition, one image was added to those used in the previous two experiments. For the adult animal condition, one image was added and two images were replaced because they were rated as fairly cute in the previous experiments. For the neutral object condition, eight neutral images were selected from the International Affective Picture System [38] (*M* valence = 5.1 and *M* arousal = 2.7 on 9-point scales according to normative ratings).

Participants were asked to indicate whether a stimulus presented on a cathode ray tube screen contained the letter *H* or the letter *T* by pressing the left or right key on a response pad as quickly and as accurately as possible. Figure 2B shows examples of the stimuli. Each stimulus was a larger letter composed of smaller letters. Each vertical and horizontal line of a global letter consisted of closely spaced local letters. The global and local letters included *F*, *H*, *L*, or *T*. There were eight types of letters. Half of them were global targets (two global *H*s with local *F*s or *L*s and two global *T*s with local *F*s or *L*s), whereas the other half were local targets (two local *H*s in global *F*s or *L*s and two local *T*s in global *F*s or *L*s). The three conditions were run in blocks. In each condition, the participants viewed each of the eight images for 3 s and then performed the eight types of trials in a random order. A stimulus letter disappeared upon pressing a key, and the next letter appeared 250 ms thereafter. After every eight trials, a fixation cross was presented for 500 ms before the next image. Consequently, each block contained 32 global-target and 32 local-target trials. After completing all conditions, the participants were asked to rate the 24 images based on the same scales used in the previous experiments.

RTs were logarithmically transformed. Trials with error responses (2.6% of the total trials) or with RTs over 3 standard deviations from the mean (0.4% of the trials) for each stimulus were removed from averaging. A multivariate ANOVA with within-participants factors of condition (baby animals, adult animals, and neutral objects) and attentional focus (global vs. local) and a between-participants factor of gender was performed on RTs. Error rates were not analyzed statistically because they were uniformly low.

### Results and Discussion

The mean scores of picture ratings are shown in Table 1. The ratings of all scales differed across conditions, *F*s(2, 33) = 144.96, 142.25, 73.70, and 13.45; *p*s <0.001, η_p_^2^s = 0.90, 0.90, 0.82, and 0.45, for cuteness, infantility, pleasantness, and excitement, respectively. The results of post hoc comparisons are summarized as follows. The images of baby animals were rated as cuter (*M* = 5.0 vs. *M* = 3.9) and more infantile (*M* = 5.0 vs. *M* = 2.5) than the images of adult animals. In contrast to the previous experiments with a between-participants design, the images of baby animals were rated as more pleasant (*M* = 4.7 vs. *M* = 4.1) than the images of adult animals. Excitement ratings did not differ between the images of baby and adult animals (*M* = 3.0 vs. *M* = 2.8). The images of neutral objects were rated as the least cute, least infantile, least pleasant, and least exciting (*M*s = 2.6, 2.1, 3.0, and 1.9, respectively). As in Experiment 2, women gave higher cuteness ratings than men to all types of stimuli (*M* = 4.0 vs. *M* = 3.6), *F*(1, 34) = 8.86, *p* = 0.005, η_p_^2^ = 0.21.

**Figure 4:**
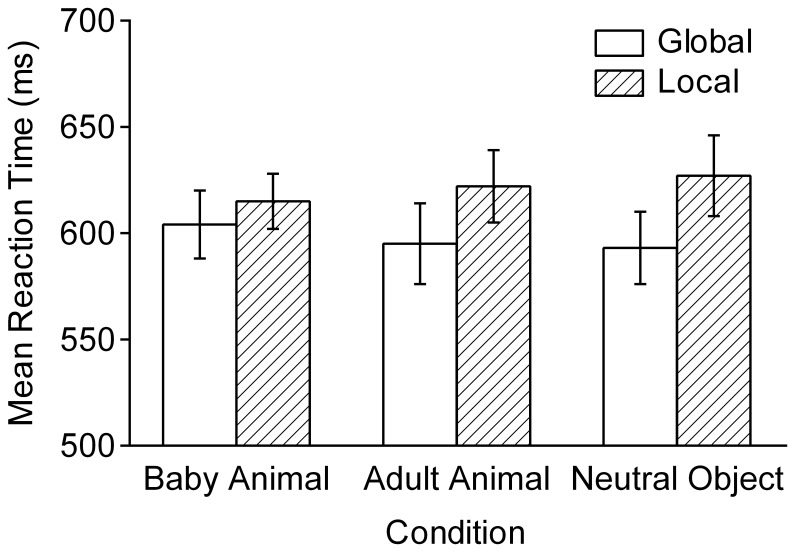
Mean reaction times in the global–local letter task (Experiment 3). Participants performed the task in three blocks during which images of baby animals, adult animals, and neutral objects were presented every eight trials (*N* = 36). The error bars indicate the standard errors of means.

Figure 4 shows the mean RTs for global and local targets in the letter task. A Condition×Attentional Focus×Gender ANOVA showed no significant main effect of the condition, *F* <1. The main effect of attentional focus was significant, *F*(1, 34) = 9.70, *p* = 0.004, η_p_^2^ = 0.22, showing typical global precedence effect. However, this effect tended to differ across conditions, *F*(2, 33) = 2.89, *p* = 0.070, η_p_^2^ = 0.15 for the Condition×Attentional Focus interaction. After viewing images of baby animals, no significant global precedence effect was found, *t*(35) = 1.54, *p* = 0.132, *r* = 0.08. In contrast, the RT difference was significant after viewing the images of adult animals and neutral objects, *t*s(35) = 2.82 and 3.45, *p*s = 0.008 and 0.001, *r*s = 0.14 and 0.16, respectively. To test whether viewing baby animal images reduced the global precedence effect, a contrast-coded ANOVA was conducted on the local-minus-global RT difference score. The assigned weights were +2 for the baby animal conditions and −1 for the adult animal condition and the neutral condition. The contrast was significant, *F*(1, 34) = 5.94, *p* = 0.020, η_p_^2^ = 0.15, which shows that the RT difference between global and local targets was smaller in the baby animal condition than in the other conditions. No significant gender-related effects on RTs were found.

The results show that viewing cute images reduces the global precedence effect as compared to viewing the other, less cute images. Because the mean RT did not differ across conditions, the effect was not due to the general improvement of performance. Gable and Harmon-Jones [18], [34] proposed that positive affect with high approach motivation (e.g., desire) narrows the breadth of cognition and attentional focus, whereas positive affect with low approach motivation (e.g., amusement) broadens it. The present study replicated and extended the finding of Gable and Harmon-Jones that viewing images of baby animals reduces the global precedence effect (Study 3 in Ref. [18]) by showing that the reduction was caused by viewing cute animals, not by viewing animals in general. The narrowed attention may be beneficial to performance on tasks that require carefulness in the motor and perceptual domains, such as the tasks used in the first two experiments.

According to Gable and Harmon-Jones’ account [18], [34], the intensity of approach motivation induced by cute images is the key to the performance improvement in tasks that require carefulness. However, this account appears to disagree with the result of Experiment 2 that performance was not improved after viewing images of pleasant foods, which are likely to elicit approach motivation. Two explanations are possible. The first explanation is that food images used in the present study did not induce high approach motivation. It is known that the viewer’s expectation to consume the foods affects the intensity of approach motivation induced by food stimuli [18]. As we did not give any indication to eat them, food images could have weak or no effect on approach motivation. Cute images, on the other hand, could serve as a powerful stimulant for approach motivation in wider contexts. The second explanation is that the feeling of cuteness induces a specific behavioral tendency that is not directly linked to the general level of approach motivation. In fact, the three types of images used in Experiment 2 did not differ in excitement ratings. The mean rating scores were relatively low (from 1.9 to 2.1 on a 6-point scale). Griskevicius, Shiota, and Neufeld [27] adopted a functional evolutionary approach and proposed that discrete positive emotions are linked to certain types of processing. They examined the effect of different positive emotions on the processing of strong and weak persuasive messages. The participants were told to elicit one of six examples of positive emotions (anticipatory enthusiasm, contentment, attachment love, amusement, awe, and nurturant love) by recalling experiences or by reading descriptions. The participants were then asked about their attitude toward a persuasive message. The results show that positive emotions of anticipatory enthusiasm, attachment love, and amusement facilitate the acceptance of weak messages, whereas positive emotions of awe and nurturant love reduce persuasion by weak messages. According to Griskevicius et al. [27], nurturant love is the feeling of love and concern for another’s well-being, which typically occurs when seeing infants, small children, and baby animals. In this sense, nurturant love is similar to the feeling elicited by the cute images used in the present study. This type of experience can be seen as an emotion [39]. Griskevicius et al. [27] assumed that nurturant love motivates caregiving behavior that exhibits close attention to the target’s needs and vigilance against possible threats to the target, and thus increases the systematic processing of the environment. The finding that viewing cute images narrowed the breadth of attentional focus can also be explained in terms of the facilitated tendency toward systematic processing.

The current findings are not conclusive enough to decide which account is more appropriate to explain the effect of viewing cute images: the approach motivation account of Gable and Harmon-Jones [18], [34] or the nurturant love account of Griskevicius et al. [27]. So far as the present study is concerned, these hypotheses may not be mutually exclusive in that nurturant love is inseparable from the desire to approach. One important consequence of both hypotheses is that viewing cute things affects behavioral tendencies but not general performance levels. In Experiment 3, the overall RT was not reduced after viewing cute images. If viewing cute images narrows the breadth of attentional focus and facilitates systematic processing, it may degrade performance in tasks that require broader attentional focus and heuristic processing. Further research is needed to explore this hypothesis.

## General Discussion

This study examined the effect of viewing cute images on subsequent performance in unrelated tasks. The images of baby animals were rated as cuter and more infantile than the images of adult animals in all the three experiments. When the participants rated the images of both baby and adult animals (Experiment 3), the former were rated as more pleasant than the latter. In the first two experiments, viewing cute images improved performance on tasks that required carefulness. This effect was found in a fine motor dexterity task that was related to helping others (Experiment 1) and in a non-motor visual search task that was irrelevant to caregiving or social interaction (Experiment 2). The improvement was associated with either a decrease or an increase in performance speed, depending on the nature of the task. Experiment 3 showed that viewing cute images narrows the breadth of attentional focus and reduces the global precedence effect in a subsequent task.

The results replicated and extended the result of Sherman et al. [16] that viewing cute images has a positive effect on behavioral performance in tasks that require carefulness. The effect occurred not only in the motor domain but also in the perceptual domain. Sherman et al. [16] explained the result in terms of the embodied cognition perspective, which holds that the tenderness elicited by cute images is more than just a positive affective feeling state, but it can make people more physically tender in their motor behavior. The present study shows that perceiving cuteness not only improves fine motor skills but also increases perceptual carefulness. While Sherman and Haidt [17] proposed that cuteness cues motivate social engagement, the current findings show that the effect of cuteness goes beyond the tasks that suggest social interaction. This study does not deny the view that cuteness is related to embodied cognition and sociality motivation. Rather, this study provides further evidence that perceiving cuteness exerts immediate effects on cognition and behavior in a wider context than that related to caregiving or social interaction.

There are several limitations in this study. First, the psychophysiological state underlying the feeling of cuteness has to be explored. Shiota, Neufeld, Yeung, Moser, and Perea [40] reported that viewing baby animals was associated with increased heart rate and increased respiratory rate, implying increased arousal. Sherman et al. [16] also found that the heart rate increased from the baseline as participants viewed baby animals. However, this finding was not replicated in their second experiment. The details of the psychophysiological responses to cute stimuli remain unclear and need to be determined in future investigations. These data would be helpful to decide which account is more appropriate to explain the current findings: the approach motivation account that is based on a dimensional model of emotion [18] or the nurturant love account that assumes a discrete, specific emotion [27]. Second, the lack of gender differences is worth re-examining with a larger sample. Sherman et al. [16] also did not find a significant gender effect on performance in the operation task. Women often give a better conscious appraisal toward infants than men [21], [23]. In the present study, women gave higher cuteness ratings than men, regardless of the type of image (Experiments 2 and 3). However, the gender differences may not be directly linked to an overt behavior or may not be strong enough to produce behavioral differences [8], [23]. The sensitivity to cuteness cues also varies among women depending on their hormonal states [22]. In addition, it has been suggested that the attitudes and responsiveness toward infants and babies differ between cultures [41]. Japan’s culture accepts and appreciates childishness at the social level [1]. Thus, gender differences might be even reduced in Japanese participants as compared with Western counterparts. Besides gender, individual differences in personality (e.g., trait empathy) and preference for images are worth examining in future research as a moderator variable between viewing cute images and performance improvement.

*Kawaii* things not only make us happier, but also affect our behavior. This study shows that viewing cute things improves subsequent performance in tasks that require behavioral carefulness, possibly by narrowing the breadth of attentional focus. This effect is not specific to tasks related to caregiving or social interaction. For future applications, cute objects may be used as a facile emotion elicitor. Cute features not only make objects more user friendly and approachable [3], but also induce careful behavioral tendencies in the users, which is beneficial in specific situations, such as driving and office work.
